# Supplementary material for: Perseus: lineage-aware refinement of Kraken2 taxonomic classification for long read metagenomes
Source: Bioinformatics. 2026 Sep 16;42(10):btag687. doi: 10.1093/bioinformatics/btag687 (PMC13633669; doi:10.1093/bioinformatics/btag687)
Supplement: Supplementary file 1 [file btag687_supplementary_data.pdf]

Supplementary Material

Perseus: Lineage-Aware Refinement of Kraken2 Taxonomic Classification for Long Reads

Supplementary Methods

Rule-based confidence baselines

To assess whether the performance gains from Perseus can be explained by simple heuristics derived from the Kraken2 output, we constructed seven confidence metrics based on the distribution of k-mer assignments reported by Kraken2. For each metric, reads were filtered using a range of threshold values and performance was evaluated across call-rates.

Support fraction

The support fraction measures the proportion of classified k-mers assigned directly to the predicted taxon:

$$\text{SupportFraction} = \frac{N_{\text{pred}}}{N_{\text{classified}}}$$

where N_{pred} is the number of k-mers assigned to the predicted taxon and $N_{\text{classified}}$ is the total number of classified k-mers

Spatial spread

Spatial spread measures how the supporting evidence is distributed across the sequence:

$$\text{Spread} = \frac{B_{\text{pred}}}{B_{\text{total}}}$$

where B_{pred} is the number of 1kb bins of the sequence contain support for the predicted taxon and B_{total} is the total number of bins in the sequence.

Lineage consistency

Lineage consistency measures the fraction of classified k-mers assigned to taxa that are within the predicted lineage:

$$\text{LineageConsistency} = \frac{N_{\text{ancestor}} + N_{\text{descendant}}}{N_{\text{classified}}}$$

where N_{ancestor} is the number of k-mers assigned to ancestors and $N_{\text{descendants}}$ is the number of k-mers assigned to descendants of the predicted taxon.

Lineage purity

Lineage purity measures the proportion of lineage-consistent evidence assigned directly to the predicted taxon:

$$\text{LineagePurity} = \frac{N_{\text{pred}}}{N_{\text{ancestor}} + N_{\text{descendant}}}$$

Descendant fraction

The descendant fraction measures the proportion of classified k-mers that are assigned to descendants of the predicted taxon:

$$\text{DescendantFraction} = \frac{N_{\text{descendant}}}{N_{\text{classified}}}$$

where $N_{\text{descendant}}$ is the number of k-mers assigned to descendants of the predicted taxon.

Out-of-lineage fraction

The out-of-lineage fraction measures the proportion of evidence inconsistent with the predicted lineage:

$$\text{OutOfLineageFraction} = 1 - \text{LineageConsistency}$$

Taxon entropy

Taxonomic uncertainty was measured using the Shannon entropy:

$$\text{Entropy} = -\sum_i p_i \log_2 p_i$$

where $p_i = \frac{N_i}{\sum_j N_j}$. Higher values indicate evidence distributed across many taxa, whereas lower values indicated concentrated support.

Random forest baseline

To assess whether the performance gains from Perseus come from its convolutional architecture rather than its input representation, we trained a random forest classifier using the same features provided as input to Perseus. Each sequence was represented as a collection of bins, where each bin contained the same lineage-based feature vector used by Perseus.

The random forest model was trained to predict whether each individual bin was consistent with the correctness of the Kraken2 taxonomic assignment. During inference, the classifier produced a probability for every bin in a sequence. The sequence-level confidence score was computed as the arithmetic mean of the predicted probabilities across all bins of the sequence. Varying the threshold for binary classification of whether a classification should be accepted or rejected generated a call-rate-performance curve similar to those reported for Perseus.

Supplementary Figures

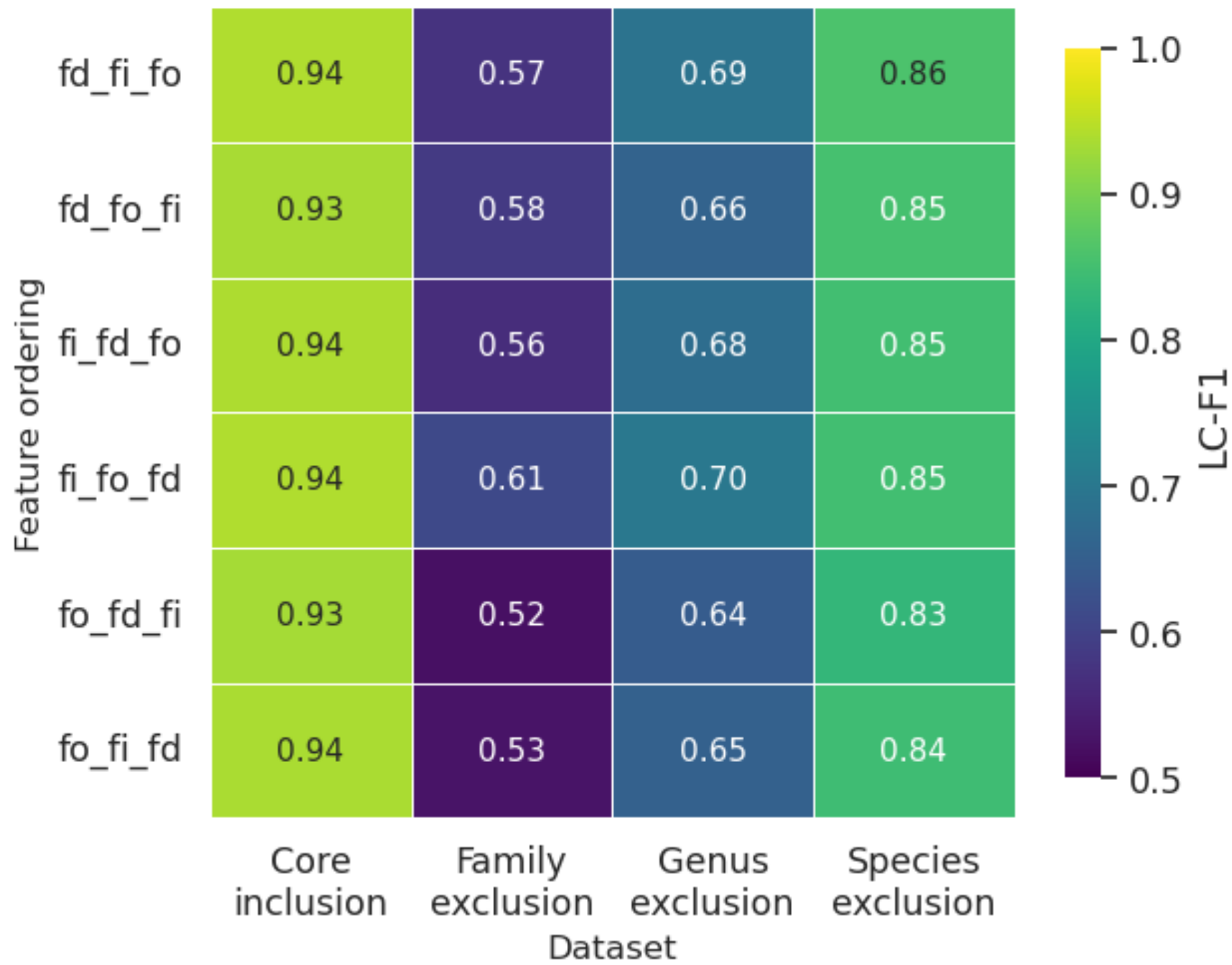

Figure S1: *Effect of feature group ordering on Perseus performance.* The three feature groups comprising of in-lineage (fi), out-of-lineage (fo) and descendant (fd) feature vectors, were permuted in all six possible orders. Perseus was retrained from scratch for each permutation. Heatmap values show the lineage-correct F1-score (LC-F1) for each evaluation dataset. Performance remained broadly consistent across feature orderings with modest variation across more challenging exclusion datasets, showing the Perseus is not dependent on specific arrangements of the feature groups.

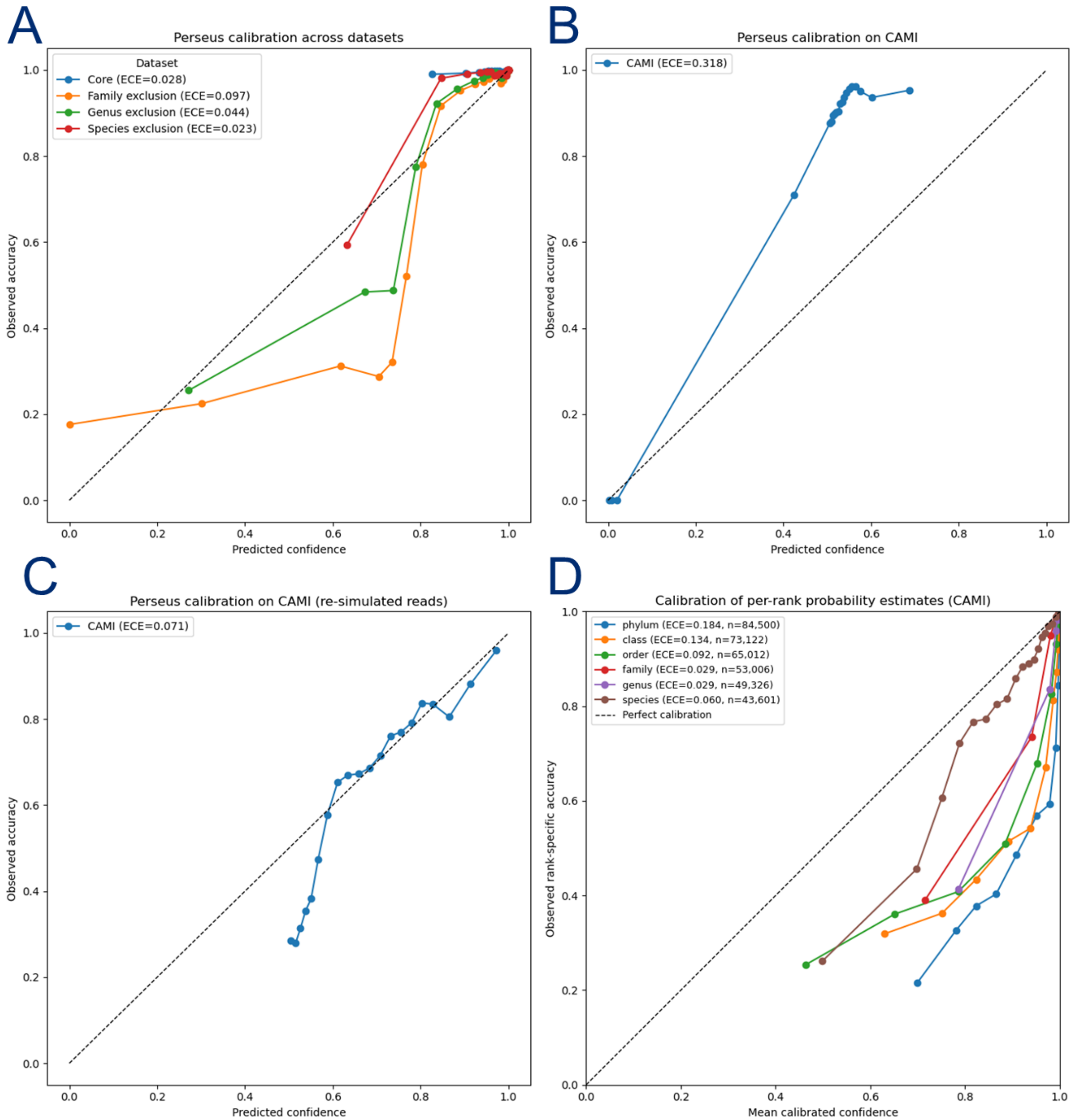

Figure S2: *Calibration of Perseus confidence estimates.* (A) Reliability diagrams for the final calibrated confidence estimates across the core inclusion and species-, genus-, and family-exclusion datasets. Predicted confidence closely matches observed accuracy, with expected calibration error (ECE) ranging from 0.023 to 0.097, demonstrating good calibration on simulated evaluation datasets. (B) Reliability diagram for the CAMI plant-associated read dataset. Although Perseus remains discriminative, the model is systematically underconfident, reflecting a distribution shift relative to the training data. (C) Reliability diagram for reads simulated from the same CAMI reference genomes using the training data generation pipeline. Calibration improves substantially, indicating that the reduced calibration in panel B is primarily attributable to differences in sequencing characteristics, rather than the underlying genomes. (D) Reliability diagrams for the independently calibrated per-rank probability estimates on the CAMI dataset. Each output head was calibrated separately. The resulting probabilities generally follow the corresponding empirical rank-specific accuracies, with the strongest calibration observed a lower taxonomic ranks (i.e. family, genus, species). The dashed line in all panels denotes perfect calibration.

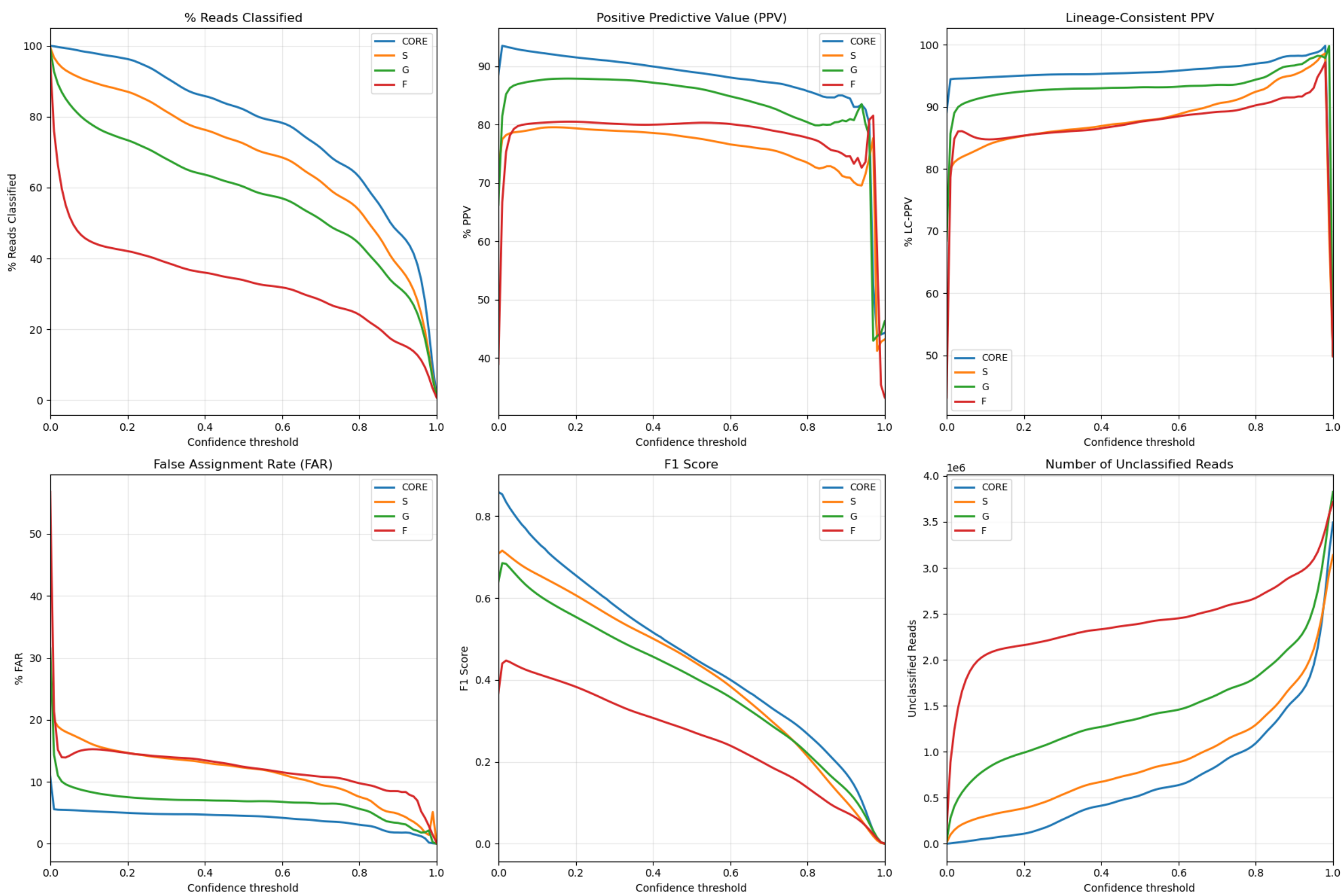

Figure S3: *Performance of Kraken2 across confidence thresholds in the inclusion/exclusion evaluation datasets.* Metrics shown include (top row) percent reads classified, positive predictive value (PPV), and lineage-consistent PPV; (bottom row) false assignment rate (FAR), F1 score, and number of unclassified reads. CORE denotes the inclusion dataset; S, G, and F denote species-, genus-, and family-exclusion datasets. As confidence increases, call rate decreases while precision improves, illustrating the sensitivity–precision tradeoff under increasing taxonomic novelty.

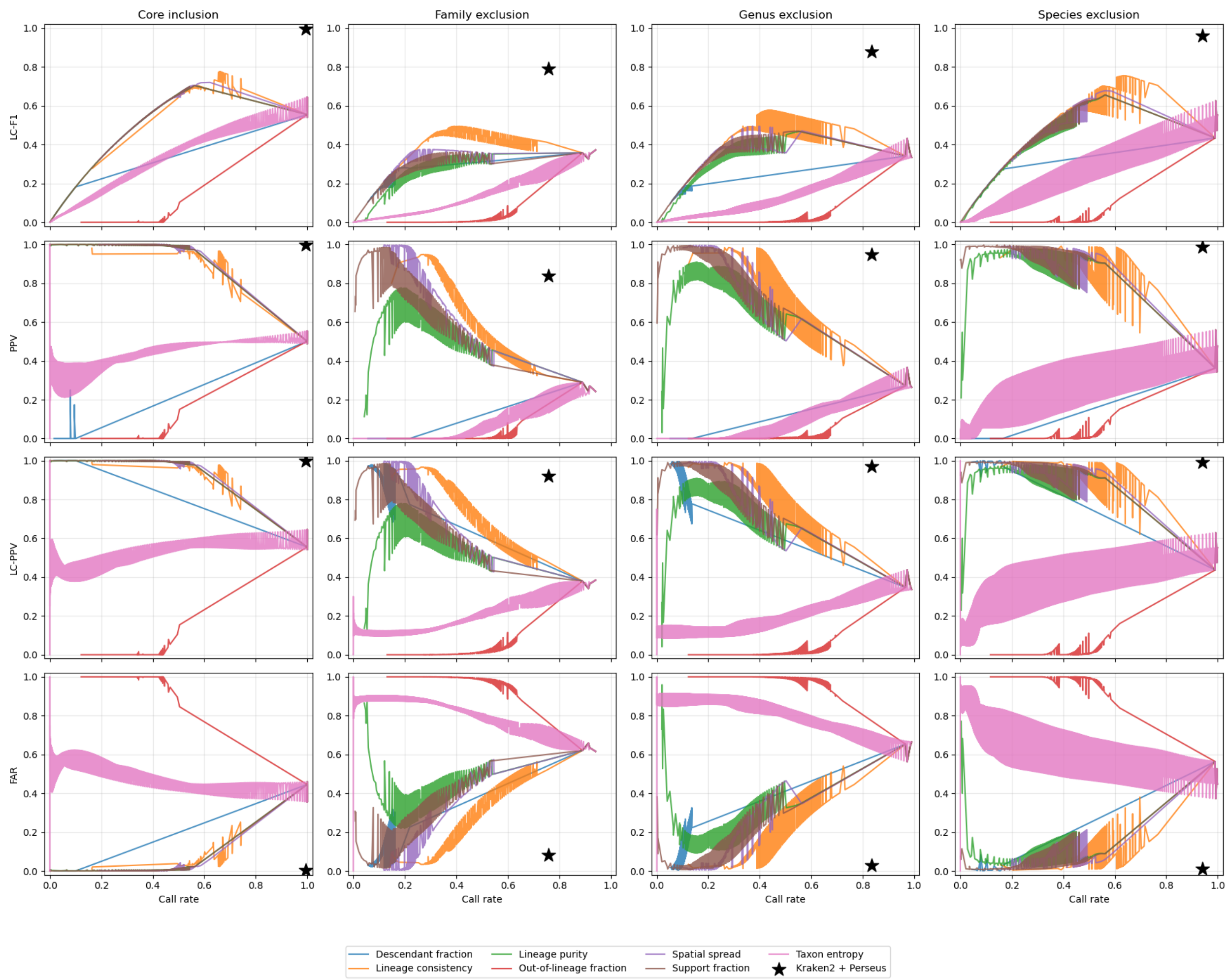

Figure S4: *Comparison of rule-based baseline filters and Perseus across inclusion/exclusion evaluation datasets.* Curves show the performance obtained by thresholding individual handcrafted features derived from Kraken2 k-mer assignments. Stars indicate the performance of Perseus filtering Kraken2 classifications. Columns correspond to the inclusion and exclusion datasets. Rows show lineage-correct F1 score (LC-F1), false assignment rate (FAR), positive predictive value (PPV), and lineage-correct PPV (LC-PPV). None of the baselines consistently match Perseus across all metrics and datasets.

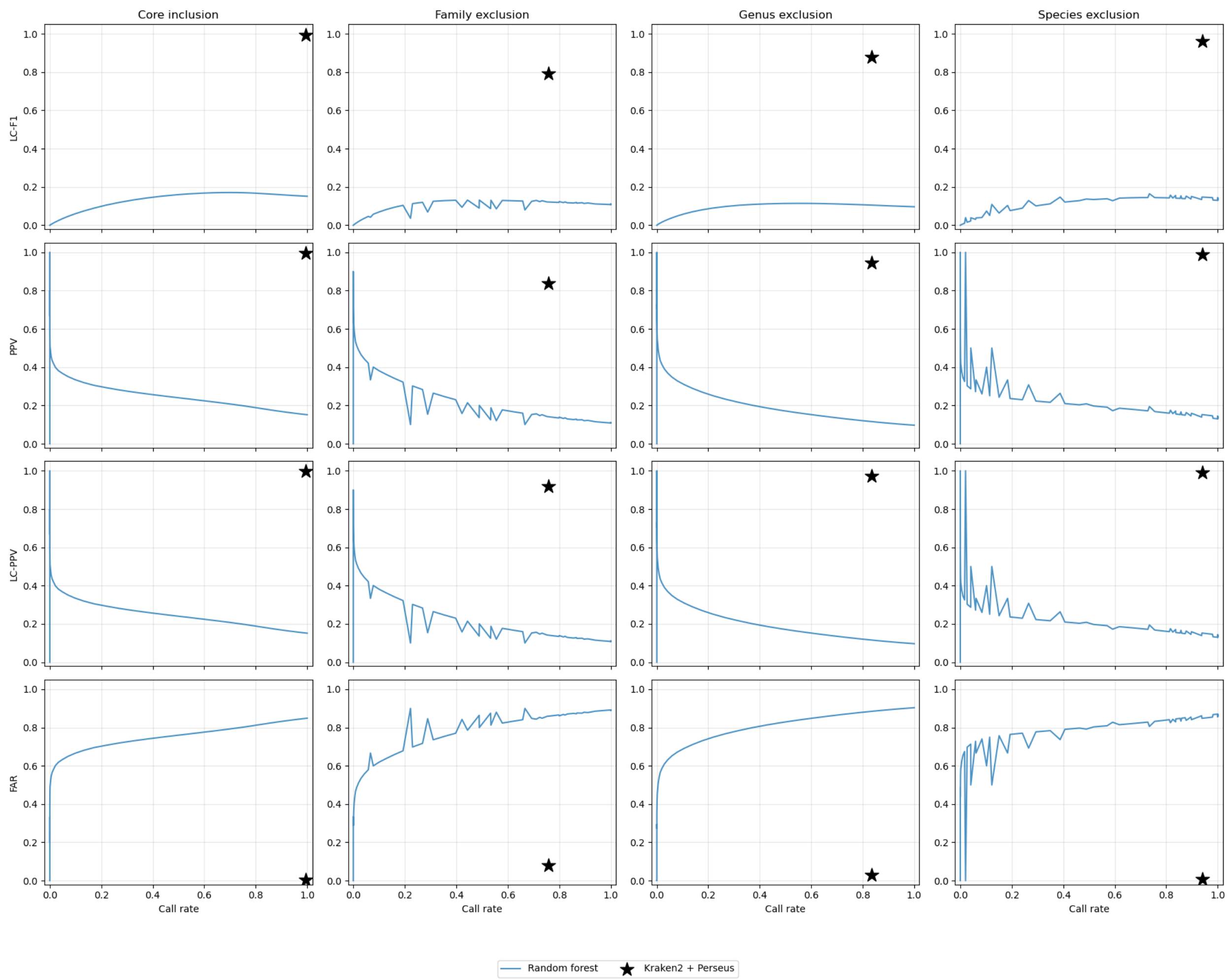

Figure S5: *Comparison of Random Forest baseline and Perseus across inclusion/exclusion evaluation datasets.* Curves show classification performance of the Random Forest baseline as the decision threshold is varied, while stars indicate the performance of Perseus filtering Kraken2 classifications. Columns correspond to the inclusion and exclusion datasets. Rows show lineage-correct F1 score (LC-F1), false assignment rate (FAR), positive predictive value (PPV), and lineage-correct PPV (LC-PPV). Perseus achieves higher LC-F1, PPV, and LC-PPV while maintaining a substantially lower FAR than the baseline.

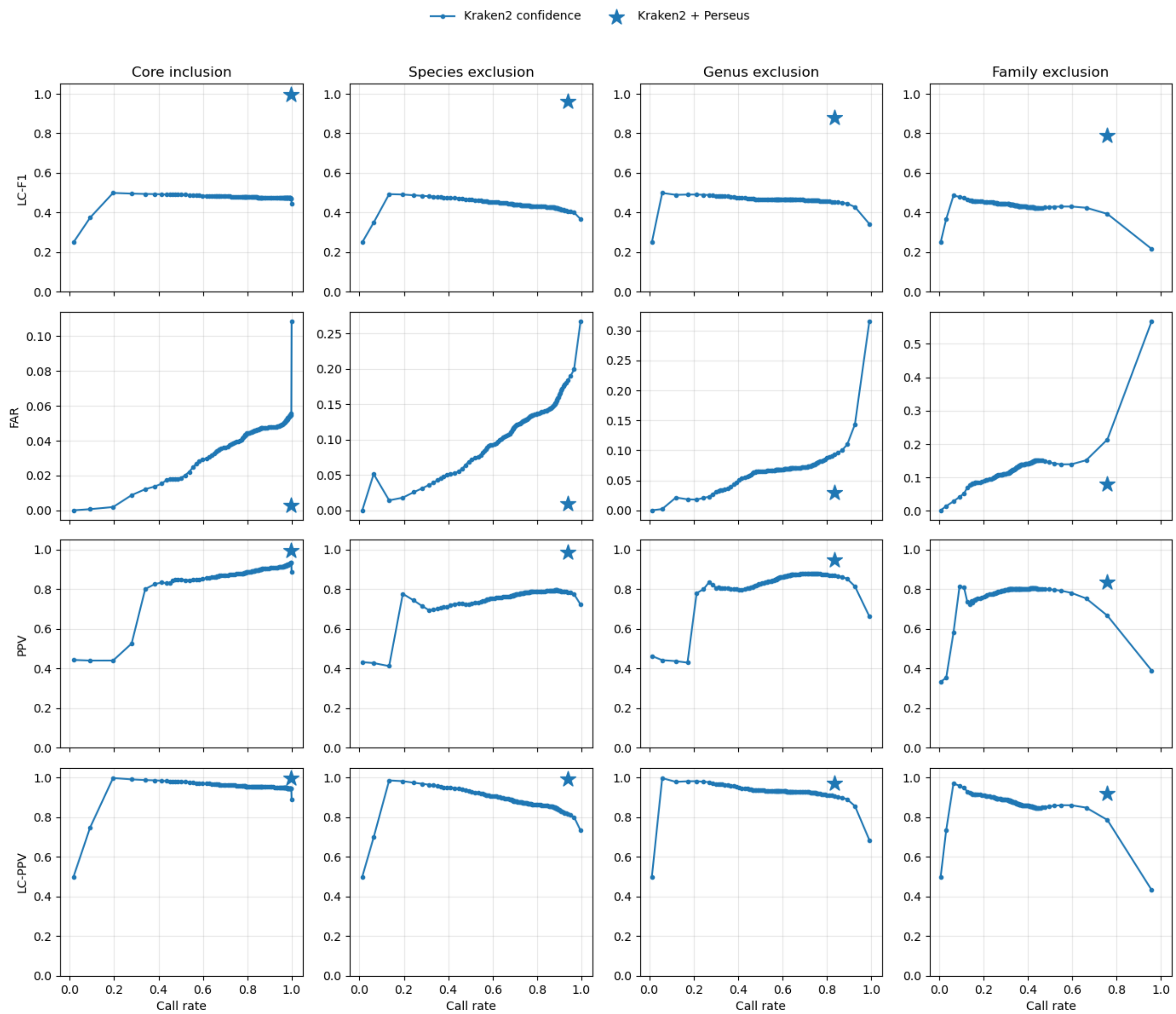

Figure S6: *Comparison of Kraken2 confidence thresholding and Perseus across inclusion/exclusion evaluation datasets.* Curves show classification performance from varying the Kraken2 confidence threshold, while stars indicate the performance of Perseus filtering Kraken2 classifications. Columns correspond to inclusion and exclusion datasets. Rows show lineage-correct F1 score (LC-F1), false assignment rate (FAR), positive predictive value (PPV), and lineage-correct PPV (LC-PPV). Perseus achieves substantially higher LC-F1, PPV, and LC-PPV whilst maintaining a far lower FAR than confidence thresholding alone under all conditions.

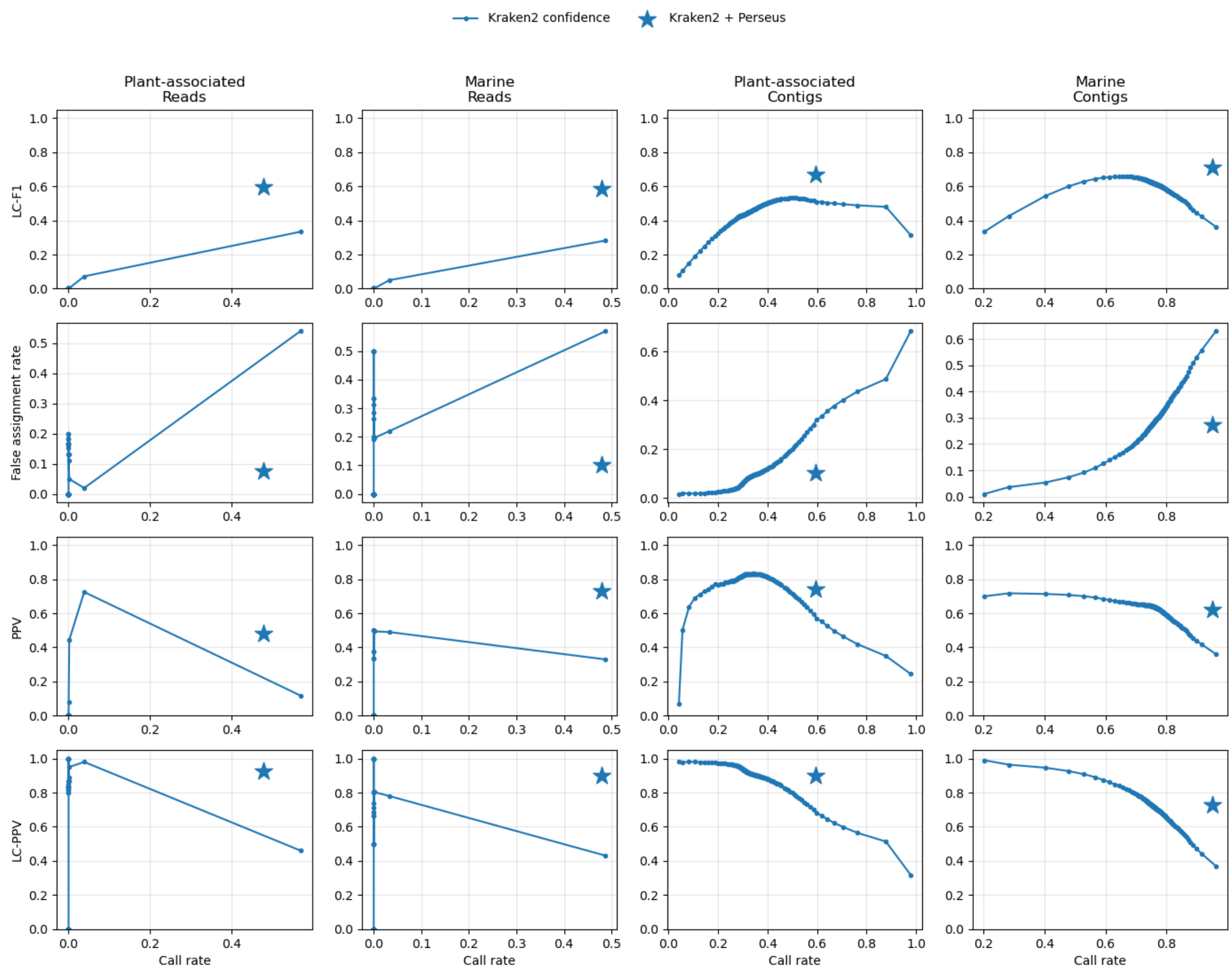

Figure S7: *Comparison of Kraken2 confidence thresholding and Perseus on CAMI read and contig benchmarks.* Curves show classification performance from varying the Kraken2 confidence threshold, while stars indicate the performance of Perseus filtering Kraken2 classifications. Columns correspond to CAMI plant-associated and marine datasets evaluated on reads and assembled contigs. Rows show lineage-correct F1 score (LC-F1), false assignment rate (FAR), positive predictive value (PPV), and lineage-correct PPV (LC-PPV). Across both reads and contigs, Perseus substantially higher LC-F1, PPV, and LC-PPV whilst maintaining a far lower FAR than confidence thresholding alone at comparable call rates.

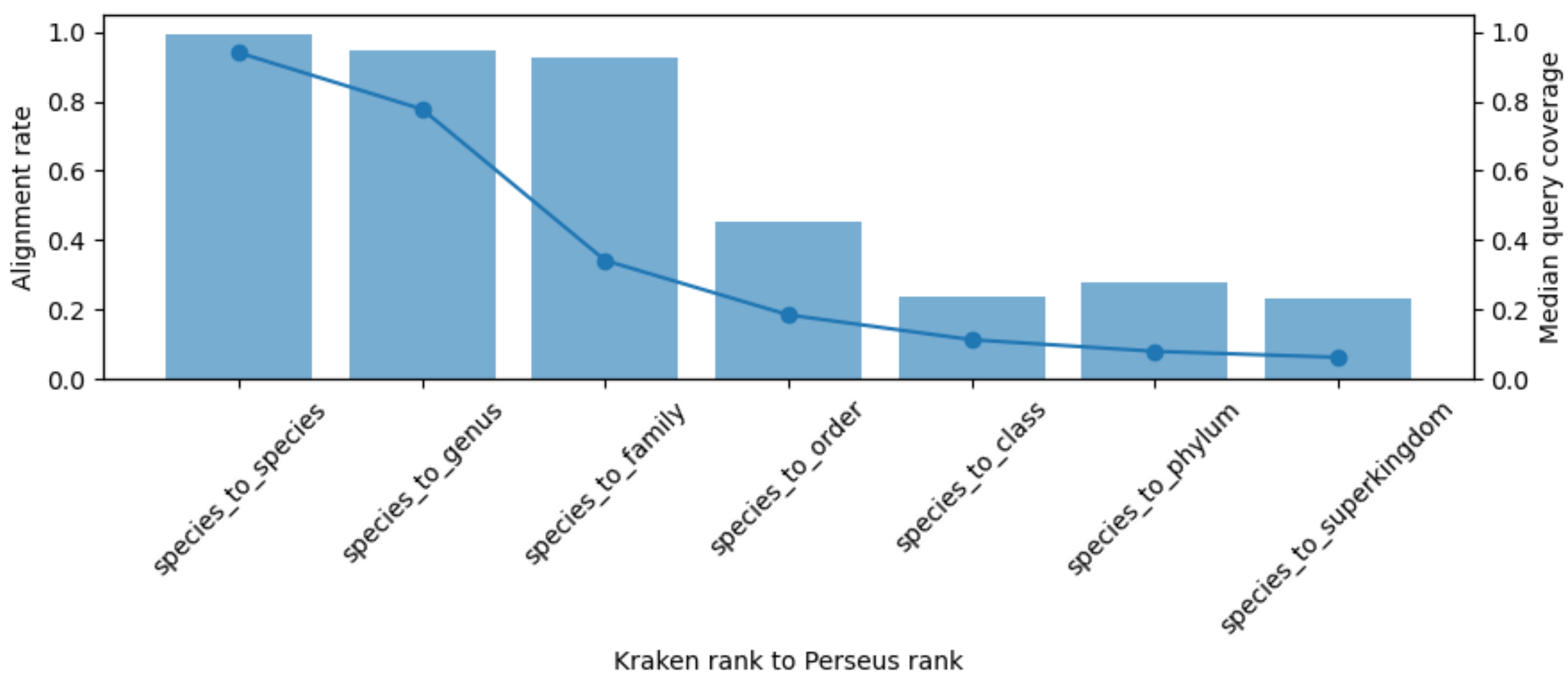

Figure S8: *Alignment performance and query coverage as a function of rank back-off from Kraken to Perseus on the German forest microbiome.* Bars show the proportion of reads that successfully aligned to the reference (alignment rate on the left y-axis), while the overlaid line shows the median query coverage among aligned reads (right y-axis). Categories on the x-axis denote the original Kraken assignment at species level and the corresponding Perseus back-off rank (species through superkingdom). As classifications are progressively backed off to higher taxonomic ranks, both alignment rate and median query coverage decrease.

Supplementary Tables

Dataset	Method	Call rate	PPV	LC-PPV	FAR	F1
Core Inclusion (n=10,563,799)	Kraken2	**0.999**	0.875 ± 0.010	0.884 ± 0.006	0.116 ± 0.006	$\mathbf{0.900 \pm 0.020}$
	Kraken2 + Perseus	0.940 ± 0.020	$\mathbf{0.997 \pm 0.001}$	$\mathbf{0.997 \pm 0.001}$	$\mathbf{0.003 \pm 0.001}$	0.881 ± 0.014
Family exclusion (n=12,781,727)	Kraken2	$\mathbf{0.916 \pm 0.010}$	0.387 ± 0.011	0.438 ± 0.010	0.562 ± 0.010	$\mathbf{0.491 \pm 0.013}$
	Kraken2 + Perseus	0.550 ± 0.015	$\mathbf{0.969 \pm 0.006}$	$\mathbf{0.982 \pm 0.004}$	$\mathbf{0.018 \pm 0.004}$	0.462 ± 0.026
Genus exclusion (n=15,313,330)	Kraken2	$\mathbf{0.978 \pm 0.005}$	0.529 ± 0.027	0.551 ± 0.026	0.449 ± 0.026	$\mathbf{0.659 \pm 0.021}$
	Kraken2 + Perseus	0.700 ± 0.045	$\mathbf{0.987 \pm 0.003}$	$\mathbf{0.992 \pm 0.002}$	$\mathbf{0.008 \pm 0.002}$	0.592 ± 0.022
Species exclusion (n=13,393,884)	Kraken2	$\mathbf{0.995 \pm 0.002}$	0.759 ± 0.025	0.770 ± 0.023	0.230 ± 0.023	$\mathbf{0.836 \pm 0.017}$
	Kraken2 + Perseus	0.807 ± 0.025	$\mathbf{0.995 \pm 0.002}$	$\mathbf{0.996 \pm 0.002}$	$\mathbf{0.004 \pm 0.002}$	0.774 ± 0.039
CAMI II (reads) marine (n=2,686,557)	Kraken2	**0.487**	0.330	0.431	0.569	**0.240**
	Kraken2 + Perseus	0.481	**0.732**	**0.897**	**0.103**	0.238
CAMI II (contigs) marine (n=394,302)	Kraken2	**0.998**	0.421	0.428	0.572	0.586
	Kraken2 + Perseus	0.949	**0.621**	**0.728**	**0.271**	**0.593**
CAMI II (reads) plant-associated (n=1,691,143)	Kraken2	**0.568**	0.118	0.461	0.539	0.586
	Kraken2 + Perseus	0.477	**0.479**	**0.924**	**0.272**	**0.593**
CAMI II (contigs) plant-associated (n=304,205)	Kraken2	**0.979**	0.244	0.318	0.682	**0.355**
	Kraken2 + Perseus	0.595	**0.740**	**0.898**	**0.102**	0.295

Table S1: *Effect of Perseus filtering on taxonomic classification performance across simulated inclusion/exclusion and CAMI II datasets.* Performance metrics are reported for baseline Kraken2 classifications and after applying Perseus filtering. The inclusion/exclusion datasets were generated with multiple independent simulation runs so the metrics contain mean and standard deviation. Perseus consistently increases precision (PPV) and lineage-consistent precision (LC-PPV) while substantially reducing the false assignment rate (FAR), at the cost of a moderate reduction in call rate due to conservative abstention. Improvements are especially pronounced in exclusion datasets and long-read assemblies (contigs), showing that Perseus effectively suppresses spurious fine-rank assignments while preserving confident higher-rank classifications. The low call rate and F1 scores observed for CAMI II read-level evaluations reflect the unusually short read lengths in this dataset, which fall outside the typical operating regime of long-read taxonomic classification. Bolded values indicate the better-performing method for each metric within a dataset.

Dataset	Species	Genus	Family	Order	Class	Phylum	Superkingdom
Core inclusion	88.07	11.37	0.28	0.23	0.04	0.01	0.00
Species exclusion	76.18	18.02	2.37	2.14	0.45	0.15	0.69
Genus exclusion	58.99	6.14	9.02	13.88	6.39	1.39	4.18
Family exclusion	46.09	4.98	0.45	17.05	15.62	4.10	11.71

Table S2: *Distribution of final predicted taxonomic ranks after filtering with Perseus.* Values represent the percentage of predictions assigned to each canonical taxonomic rank after Perseus post-processing. As higher taxonomic ranks are excluded from the reference database, Perseus backs off to broader lineage-consistent assignments rather than retaining unsupported species-level predictions.

Dataset	Same rank	Back off 1 rank	Back off 2 ranks	Back off ≥3 ranks
Core inclusion	90.90	8.60	0.26	0.23
Species exclusion	77.04	17.31	2.48	3.13
Genus exclusion	59.94	7.10	9.78	22.99
Family exclusion	47.68	6.58	3.15	42.13

Table S3: *Frequency of taxonomic rank transitions after filtering with Perseus.* Values represent the percentage of predictions whose assigned taxonomic rank remained unchanged or was reassigned to a less specific rank relative to the original Kraken2 prediction. Perseus preserves the original taxonomic rank for most predictions in the core inclusion dataset and progressively backs off assignments only as higher taxonomic ranks are absent from the reference database.

Method	Reads	CPU cores	GPU	Peak RAM (GB)	Wall time (hh:mm)
Kraken2	2,735,656	32	–	80.0	00:08
Perseus (feature extraction)	2,735,656	32	–	4.87	01:00
Perseus (CPU inference)	2,735,656	4	–	6.54	00:14
Perseus (GPU inference)	2,735,656	4	A100	7.89	00:09

Table S4: *Runtime and resource usage for Kraken2 and Perseus.* Benchmarks were run on the German forest microbiome with identical hardware. Perseus can be run with either CPU or GPU inference.
